# A cross-sectional description of social capital in an international sample of persons living with HIV/AIDS (PLWH)

**DOI:** 10.1186/1471-2458-12-188

**Published:** 2012-03-13

**Authors:** Allison Webel, J Craig Phillips, Carol Dawson Rose, William L Holzemer, Wei-Ti Chen, Lynda Tyer-Viola, Marta Rivero-Méndez, Patrice Nicholas, Kathleen Nokes, Jeanne Kemppainen, Elizabeth Sefcik, John Brion, Lucille Eller, Scholastika Iipinge, Kenn Kirksey, Dean Wantland, Puangtip Chaiphibalsarisdi, Mallory O Johnson, Carmen Portillo, Inge B Corless, Joachim Voss, Robert A Salata

**Affiliations:** 1 Clinical Research Scholar, Frances Payne Bolton School of Nursing Case Western Reserve University, 10900 Euclid Avenue, Cleveland, OH 44106-4904, USA; 2 University of British Columbia School of Nursing, Vancouver, Canada; 3 University of California, San Francisco School of Nursing, San Francisco, USA; 4 Rutgers College of Nursing, Newark, USA; 5 Yale University, New Haven, USA; 6 MGH Institute of Health Professions, Boston, USA; 7 University of Puerto Rico, San Juan, Puerto Rico; 8 Global Health and Academic Partnerships, Brigham and Women's Hospital and MGH, Institute of Health Professions, Boston, USA; 9 Hunter College, CUNY, Hunter Bellevue SON, New York, USA; 10 University of North Carolina Wilmington, Wilmington, USA; 11 Texas A&M University-Corpus Christi, Corpus Christi, USA; 12 Duke University School of Nursing, Durham, USA; 13 University of Namibia, Windhoek, Namibia; 14 Center for Nursing Research, Seton Family of Hospitals, Austin, USA; 15 Suan Sunandha Rajabhat University, Bangkok, Thailand; 16 UCSF, San Francisco, USA; 17 UCSF School of Nursing - Community Health Systems, San Francisco, USA; 18 University of Washington, Saettle, USA; 19 Department of Medicine, Division of Infectious Diseases and HIV Medicine, Case Western Reserve University, University Hospitals Case Medical Center, Cleveland, USA

**Keywords:** Social capital, HIV/AIDS, Global health, Social science

## Abstract

**Background:**

Social capital refers to the resources linked to having a strong social network. This concept plays into health outcomes among People Living with HIV/AIDS because, globally, this is a highly marginalized population. Case studies show that modifying social capital can lead to improvements in HIV transmission and management; however, there remains a lack of description or definition of social capital in international settings. The purpose of our paper was to describe the degree of social capital in an international sample of adults living with HIV/AIDS.

**Methods:**

We recruited PLWH at 16 sites from five countries including Canada, China, Namibia, Thailand, and the United States. Participants (*n *= 1,963) completed a cross-sectional survey and data were collected between August, 2009 and December, 2010. Data analyses included descriptive statistics, factor analysis, and correlational analysis.

**Results:**

Participant's mean age was 45.2 years, most (69%) identified as male, African American/Black (39.9%), and unemployed (69.5%). Total mean social capital was 2.68 points, a higher than average total social capital score. Moderate correlations were observed between self-reported physical (*r *= 0.25) and psychological condition (*r *= 0.36), social support (*r *= 0.31), and total social capital. No relationships between mental health factors, including substance use, and social capital were detected.

**Conclusions:**

This is the first report to describe levels of total social capital in an international sample of PLWH and to describe its relationship to self-reported health in this population.

## Background

Social capital is a concept that has been widely studied and discussed in public health over the past decade [1-4]. It has been defined as the "aggregate or potential resources which are linked to possession of a durable network of more or less institutionalized relationships of mutual acquaintance or recognition "(Bourdieu 1985, p248) [5,6]. In the field of HIV, scholars, clinicians, and policy-makers have expressed great interest in the concept as an explanation for trends observed among persons living with HIV/AIDS (PLWH) [7-10]. Globally, HIV/AIDS disproportionately affects the most marginalized populations, those living in poverty, the uneducated, and the socially disconnected [11-13]. Recently the paradigm that assumes poverty as the underlying factor for much of the worlds marginalized PLWH has been challenged and new evidence suggests that wealth inequity in a society is a more important factor for explaining HIV risk at the population level [3,14,15]. Viewing wealth inequity as an HIV risk factor is controversial, but has important implications for public health interventions [4,16]. Social capital may clarify the role and implications of the social environment on health outcomes among PLWH [4,16].

The relationship between social capital and health may be different among PLWH in comparison to a more general population. Globally, PLWH are highly marginalized [11-13] often diagnosed with more chronic health conditions [17-22], and decreased access to health care resources [23,24] than their counterparts in the general population. Furthermore, many PLWH experience chaotic personal environments that lead to negative health outcomes [25]. The social environment of PLWH provides the context that shapes this population's decisions about health behavior, and ultimately health outcomes. Social capital is an important component of the social environment for PLWH [26]. However, the literature lacks a basic description of social capital in an international sample of men and women living with HIV. Knowledge of the influence of social capital on health outcomes among men and women living with HIV from an international sample could inform evidence-based, public health interventions, seeking to modify social capital among PLWH and, perhaps, their overall health outcomes.

### Social capital and HIV

Research on social capital and HIV has predominantly focused on preventing HIV transmission [27]. Authors of a qualitative investigation on social capital and HIV have reported that increases in social capital appear to reduce HIV transmission risk among South Asian male immigrants to the United States [28]. Increases in both structural and cognitive social capital were also observed to change HIV risk behavior, and ultimately HIV transmission in Tanzania [29]. Additionally, HIV/AIDS prevention programs designed to increase social capital were also found to decrease HIV transmission among rural Caribbean youth [30]. This evidence suggests social capital holds promise for developing strategies to decrease HIV transmission. However, after more than a decade of inquiry, still more rigorous research is needed before definitive conclusions about the nature and usefulness of social capital as a means of HIV prevention can be drawn.

Building upon qualitative research, investigators reporting on population-based surveys of social capital and HIV prevention illustrated complex relationships between social capital and HIV transmission. Social capital has significantly predicted AIDS and other sexually transmitted diseases case rates throughout the United States [31]. Increased social capital was associated with decreased HIV prevalence among South African males, whereas in South African females higher social capital was associated with increased HIV prevalence [4]. In Zimbabwe, one aspect of social capital, participation on the local community, was associated with successful avoidance of HIV infection among women [32] and in Namibia, social capital predicted both positive and negative effects on HIV prevention behaviors [33]. Similar findings have been reported in Uganda [34] and recently, in the rural United States [35]. This evidence underscores the complex, yet important role of social capital in HIV transmission. Further, it suggests that a better general understanding of social capital and its health correlates, among PLWH around the globe may aid investigators in developing effective social capital -based, public health interventions.

Despite the substantial evidence exploring the multiple aspects of social capital and prevention of HIV transmission, many questions remain unanswered about social capital among PLWH. These gaps in the literature include both the lack of a basic description of social capital, and a description of its health correlates, in a diverse sample of PLWH. The purpose of this paper is to close these gaps and describe social capital in an international sample of men and women living with HIV/AIDS. Therefore, in this report we aim to: Describe levels of social capital in an international sample of adults living with HIV/AIDS, provide evidence for the validity of the individual-level, Social Capital Scale in this population, and determine the nature of associations between social capital, physical and psychological health, social support, and HIV status among PLWH.

## Methods

### Sample and setting

Data for this cross-sectional study come from the International Nursing Network for HIV/AIDS Research, Study V: *Exploring the Role of Self-compassion, Self-efficacy and Self-esteem for HIV-positive Individuals Managing Their Disease*. In this study, there were 16 sites from five countries and Puerto Rico. Data were collected between August, 2009 and December, 2010. Each site recruited approximately 100 participants. Participants included adults (> 18 years of age), living with HIV/AIDS, and recruited from Infectious Disease clinics and AIDS Service Organizations. Each site adhered to the common protocol and all participants gave informed consent before completing a pen and paper, cross-sectional survey [36].

After completing the survey, all data were entered into an electronic database and were de-identified. The de-identified data were sent to the coordinating center, cleaned, entered into the master database, and stored until all sites completed data collection and entry. Original data were stored at each individual site. Prior to recruitment at study sites, the Committee on the Protection of Human Subjects at the University of California, San Francisco, reviewed and approved the overall protocol and each site also received approval from their local human subjects review committees.

### Measures

The instruments used to measure the variables of interest are listed below. Social Action Theory [37-39] guided our selection of constructs, and ultimately our selection of instruments, for the study. Social Action Theory is particularly relevant to frame our understanding of individual-level social capital and health in PLWH because it considers the multi-level context in which PLWH act. We conceptualized social capital as an individual-level contextual factor that would be mediated by regulatory factors, leading to protective health actions, and ultimately health outcomes, in PLWH. The emphasis Social Action Theory places on empowerment, critical consciousness, and community capacity are important factors to consider when describing heath disparities in marginalized populations [40].

### Social capital

Measurement of social capital in the academic literature is varied and often critiqued [41,42]. The purpose of our analysis was to examine perceived relationships between social capital and individual health outcomes. Therefore, we chose to assess self-reported individual-level social capital using 31-items, from the 36-item Social Capital Scale [43-47]. This widely-used instrument measures eight subscales including: participation in the local community, social agency, feelings of trust and safety, neighborhood connections, friends and family connections, tolerance of diversity, value of life, and workplace connections; these items were used to create a total score. In our analysis, the three workplace connections items have been dropped, as well as two work-related questions that are part of the social agency dimension. This was due to low anticipated employment status, as the average unemployment rate in PLWH ranges from 62-74% [48], and with the approval and recommendation of the scale's authors. Participants were asked to rate items on a 1-4 Likert-type scale. Higher mean scores indicate more social capital. Reliability and validity of the scale have been reported as acceptable [43]. Reliability for Social Capital Scale for our study was 0.88 and ranged from 0.84 to 0.93 for all study sites.

*Demographic, HIV disease status, and criminalization of HIV was *assessed with a 20-item demographic and illness characteristics instrument. This included age, gender, race, ethnicity, education, adequacy of income, health insurance, date first learned of HIV diagnosis, current CD4 count, viral load, HIV transmission route, and general health. In addition to demographic and biomedical disease status indicators, we also assessed whether or not HIV transmission was formally criminalized. Criminalization of HIV transmission was assessed by reviewing the relevant laws and policies in the jurisdiction of residence (either state or national laws or policies) that pertained to each individual site.

*Mental Health *was measured with several instruments including SF-12 Mental Health Quality of Life subscale, CES-D Depression Scale [49,50]. Anxiety checklist of the Symptom Checklist-90-R [51], the CAGE alcohol assessment [52], and three questions on intravenous drug use in the past three months.

*Physical and Psychological Health and Social Support *were measured with three self-report questions asking participants about their current physical and psychological health condition, and their current perceived level of social support. These questions were measured on a 10-point scale (1 = very poor, 10 = excellent).

### Statistical analysis

All data were entered into a data management program and the data integrity and assumptions were checked. Analyses were conducted in Stata (version 11.2) and included descriptive statistics, exploratory factor analyses using a principle components factor analysis with oblique rotation, bivariate correlational analyses including the Pearson product-moment correlation and Spearman's rank order correlation, and multiple regression analysis. These analyses allowed us to (1) describe the average individual-level total social capital score for PLWH, (2) provide evidence for validity and reliability of the Social Capital Scale in an international sample of adults living with HIV/AIDS, and (3) examine the relationship between social capital and psychological health, psychological support, social support, and HIV disease status.

## Results

A total of (N = 1963) HIV + adults from 16 sites in 5 countries and Puerto Rico completed the cross-sectional survey. Participants' average age was 45.2 years (SD ± 9.4) and most were male (69%), African American/Black (39.9%), and had a high school diploma (38.3%). The mean year of HIV diagnosis was 1998 (SD ± 7.4 years) indicating that our sample was fairly experienced with their HIV disease. Accordingly, most were currently prescribed anti-retroviral therapy (80.4%) and reported undetectable HIV viral loads (87.4%). Additional demographic and HIV disease information are reported in Table 1.

**Table 1:** Demographic and HIV Disease Information (*n *= 1,963)

	Frequency (%)	Mean (± SD)
**Age (years)**		45.2 (9.4)

**Gender**		

Male	1,341 (69.0)	

Female	552 (28.4)	

Transgender	45 (2.3)	

**Race**		

Asian/Pacific Islander	230 (11.9)	

African American/Black	755 (39.9)	

Hispanic/Latino	400 (20.7)	

Native American	64 (3.3)	

White/Angelo	439 (22.7)	

**Education Level**		

11th grade or less	544 (28.0)	

High School or GED	746 (38.3)	

2 yrs college/AA	411 (21.1)	

4 yrs college/BS/BA	193 (9.9)	

Master's Degree or Doctorate	52 (2.6)	

**Self-Reported Income Adequacy**		

Totally inadequate	540 (28.0)	

Bare adequate	987 (51.1)	

Enough	403 (20.9)	

		

**Work for Pay**	430 (22.1)	

**Has Health Insurance**	1,354 (69.5)	

**Self-Reported HIV Indicators**		

**Year Diagnosed with HIV**		1998 (7.4)

**Prescribed Anti-Retroviral Therapy**	1,578 (80.4)	

**Has AIDS Diagnosis**	816 (42.5)	

**Undetectable Viral Load**	1,716 (87.4)	

**Viral Load for those with detectable values/mL**		29,406 (87,605)

**CD4 cells/μ1**		496.5 (360.1)

**Self-Reported HIV Transmission Method^1^**		

	Women	Men

Sex with a man with HIV	441 (22.7%)	793 (40.8%)

Sex with a woman with HIV	36 (1.9%)	411 (21.2%)

Sharing needles	140 (7.2%)	319 (16.4%)

Blood transfusion	63 (3.2%))	105 (5.4%)

Do not know	35 (1.8%)	125 (6.4%)

Mental Health Variables		

Mean Mental Health Quality of Life (^+/- ^SD)	45.19 (11.23)	44.07 (11.56)

Mean Total Depression Score (^+/- ^SD)	22.44 (11.4)	21.56 (10.9)

Mean Total Anxiety Score(^+/- ^SD)	16.88 (8.7)	18.08 (9.3)

^1: ^Participants were asked to list the possible ways they could have been infected with HIV. Responses were not mutually exclusive and participants may have listed multiple modes of transmission. Additionally, responses are presented for those identifying gender as either female or male, those reporting an additional gender category are not included.

### Social capital

**Figure 1:**
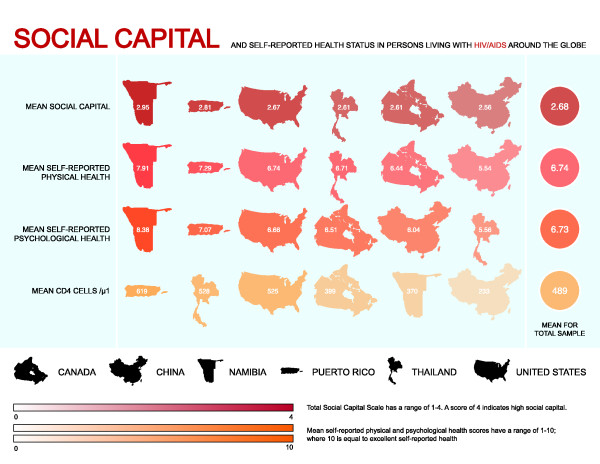
**Social Capital and Self-Reported Health Status in PLWH around the Globe**.

The mean total social capital score for all participants was 2.68 (SD ± 0.55), which indicates higher than average social capital. For individual sites the mean total social capital scores ranged from 2.56 (SD ± 0.49) in Shanghai, China to 2.95 (SD ± 0.66) in Namibia (Figure 1). The overall Cronbach's α reliability coefficient for the total Social Capital Scale was 0.88 and individual sites ranged from 0.84 (San Juan, PR and Harlington, Texas, USA) to 0.93 (Namibia). More detailed information on the mean, median and reliability statistics for the Social Capital Scale can be found in Additional file 1: Table S1. Factor analysis of the Social Capital Scale yielded a five-factor solution, explaining 65% of the variance in total social capital, with three non-loading items indicating the scale's validity. The number of items loading on each factor, the eigenvalue for each factor, and the percent variance explained by each factor are reported in Table 2. Factor loadings ranged from 0.32 to 0.80, and met the minimum 0.30 criteria for inclusion of the items [53]. We observed 11 discrepancies in the factors on which the items loaded, compared to Onyx and Bullen's original factor solution. This information is reported in Additional file 2: Table S2.

**Table 2:** Results of exploratory factor analysis: factor and factor loadings using principle components factor analysis with (Promax) oblique rotation

Factor	Items	Eigenvalue	Percent Variance Explained
Factor 1: Participation in the Local Community	6	4.59	14.8

Factor 2: Friends and Family	9	4.43	14.3

Factor 3: Tolerance and Diversity	5	3.39	12.6

Factor 4: Neighborhood Connections	4	2.87	9.3

Factor 5: Feelings of Trust and Safety	4	2.30	7.4

Nonloading Items	3	2.18	7.0

### Mental health

We observed weak correlations between mental health variables and total social capital across all study sites. The correlation coefficient for social capital and mental health quality of life was 0.02; for depression it was -0.01; for anxiety it was -0.05; for intravenous drug use it was 0.02, and for alcohol it was 0.03. We observed stronger, yet still small, effect sizes between social capital and mental health variables in Thailand and Puerto Rico. The overall correlation coefficients appear to be heavily weighted toward the U.S., perhaps due to the disproportionate number of U.S. study sites. Additional details on the relationship between mental health variables and total social capital are reported in Additional file 3 Table S3. We also examined these relationships by gender (*data not shown*), but observed no gender differences.

### Physical and psychological condition and social support

Moderate correlations were observed between self-reported physical and psychological condition, social support, and total social capital. The overall correlation between self-reported physical condition and total social capital was 0.25. For self-reported psychological condition, the correlation coefficient was 0.31. For self-reported social support, the correlation coefficient was 0.36. There was variability among the different countries, with Thailand and Puerto Rico respondents reporting weaker relationships and those in Namibia being stronger. Again, overall correlation coefficients appear heavily weighted toward U.S. results. Additional information on relationships observed between self-reported physical and psychological condition, social support, and total social capital are reported in Table 3.

**Table 3:** Bivariate correlation coefficients between social capital and self-reported health condition in HIV + Adults

Country	Sample Size	Physical Condition (*p*-value)	Psychological Condition (*p*-value)	Social Support (*p*-value)
Canada	100	0.17(0.14)	0.21(< 0.01)	0.25(< 0.01)

China	107	0.28(< 0.01)	0.21(< 0.01)	0.34(< 0.01)

Namibia	102	0.35(< 0.01)	0.35(< 0.01)	0.33(< 0.01)

Puerto Rico	100	0.06(0.61)	0.17(0.14)	0.25(0.04)

Thailand	100	0.03(0.77)	0.26(< 0.01)	0.30(< 0.01)

United States	1,454	0.25(< 0.01)	0.32(< 0.01)	0.37(< 0.01)

Total	1,963	0.25(< 0.01)	0.31(< 0.01)	0.36(< 0.01)

### HIV health status

We examined the relationship between total social capital and select HIV health status variables including CD4 count, HIV viral load, AIDS diagnosis and HIV medication adherence. No significant relationships were observed between these variables (*data not shown*).

## Discussion

Despite widespread use of social capital in research and public health interventions to decrease HIV transmission, we believe this to be the first report to describe levels of total social capital in a large, international sample of PLWH. This study helps fill a number of gaps in the literature including providing a description of levels of social capital and its correlates in an international sample of PLWH. With further development our findings can be used to help develop evidence-based, public health interventions, seeking to modify social capital among PLWH.

Our participants reported a higher than average total social capital score compared with previous research using this scale. While Bullen and Onyx (2000) do not give guidance on how to interpret the mean score, previous investigations of individual-level social capital using the Social Capital Scale have classified a mean social capital score greater than 2.5 as high social capital and anything less than 2.5 as low social capital [54]. In our study, we had an aggregate total social capital score of 2.68, with all individual sites reporting at least a mean total social capital score of 2.55. There are several plausible explanations for our higher than average social capital scores. All participants were recruited through HIV clinics or through AIDS service organizations in urban settings. By virtue of this recruitment method, participants were already engaged in their health care and possibly with related social services. This level of access, could lead them to perceive more trust in organizations, and to perceive that they have access to more social resources than other PLWH who are not as engaged in their health care. These factors could constitute a higher level of social capital. Additionally, due to anticipated low employment in our sample (22% of participants were employed) we did not include five work-related items on the original Social Capital Scale, which may have skewed our results upward. This is similar to the approach that Onyx and Bullen adopted, when addressing significant levels of unemployment in their original sample [43]. We believe this is an appropriate approach to the measurement of social capital given the sociodemographic composition of PLWH around the globe. However, taken together, our findings support recent evidence suggesting that PLWH may not be as marginalized as previously argued [3,14,15] and that the relationship between HIV and underlying structural factors in society is complex [12]. But as we discussed above, most of this literature is based on persons at risk for HIV infection not those currently living with HIV/AIDS, thus more evidence on social capital in PLWH is needed before drawing final conclusions in this regard.

The measurement of social capital is challenging [42,55]. Consistent with our aims, we assessed individual-level social capital using Onyx and Bullen's Social Capital Scale, but psychometric properties for this scale in PLWH were lacking. We observed evidence of reliability and validity of a modified Social Capital Scale in an international sample of PLWH. Our analysis of the reliability of the Social Capital Scale indicated that the scale measures a single latent construct of individual-level social capital among all sites, suggesting this scale is a reliable assessment of social capital in PLWH. However, we found differences between our sample and Onyx and Bullen's original data, when we examined the scale's validity. Our data support a five factor solution explaining 65% of the variance in total social capital, in contrast to Onyx and Bullen's original eight factor solution explaining 49% of variance [43]. These differences lie in two factors upon which the items did not load: the value of life factor and the social agency or proactivity in a social context factor. These results are surprising because the factor of social agency was one of the more explanatory factors that Onyx and Bullen found in their original scale development work. In our study, the value of life items loaded on the friends and family connections factor, suggesting that participant's perceived self-value, may be related the friends and family connections. Our observation is interesting because it harkens back to Durkheim's work on social isolation, anomie, and suicide, in that those who are more socially connected may perceive their life to be of more value and may take action to improve their health [56,57]. For PLWH, this may also translate into engaging in other risk reducing behaviors including antiretroviral therapy adherence. Another difference we observed was that items that were originally loaded on what Onyx and Bullen described as social agency, or proactivity in the social context factor, loaded on two different factors including, friends and family connections and tolerance of diversity. This may have been explained by our study samples being drawn from sites where they may perceive themselves and their peers as members of a proactive social context. They may perceive these connections as bonds between friends and families. These findings also suggest that individual-level social capital may be heavily based on the personal connections with friends and family, and the resources they provide [57]. This theory is also supported by the strong correlation between perceived social support and social capital in our sample and suggests interventions to build these connections, i.e. family or peer group-based interventions, may be helpful in facilitating behaviors to enhance the health of PLWH [58-60].

In summary, among our sample of PLWH, we observed that more of the items on the Social Capital Scale appeared to represent a subscale of friends and family connections, followed by participation in the local community, which suggests a modification to the factor structure for anyone wishing to explore the individual factors or subscales in this population in the future. However, in our analyses, we only examined relationships between social capital and health-related outcomes using the total Social Capital Scale. This alternative strategy is advantageous because it can identify factors that contribute to health outcomes among PLWH. For example, those who perceive themselves to be healthy and in possession of social capital may be empowered to collaborate with public health researchers, clinicians, and policy-makers to participate in HIV prevention, HIV treatment, and health promotion strategies [61,62]. Challenges to this strategy may be that a focus on physical health and biomedical interventions only first limits our understanding of other complex factors that are critical for individuals to access and use the social capital available to them and their community.

The moderate relationships we observed between total social capital score and self-reported physical and psychological health condition underscore the importance of perceived social resources and trust in organizations when assessing personal health. Previous investigators examining the relationship between perceived health and social capital observed similar findings in large, national samples [57]. It is possible that this observed relationship may, again, be attributed to our sampling methods, but this does not diminish the implications of these findings. Since participants were already engaged in health care, they may have had more trust in social organizations and access to necessary social resources, than persons who are less engaged with the health care system. This, in turn, gives participants an avenue from which they more easily receive information about their health and to trust that the information will be helpful and not harmful. This may increase the individual's likelihood of enacting this received health information and will allow them to more efficiently address any deviations from perceived "good health"[63]. These findings suggest a potential role for social capital in public health interventions targeting health and wellness in PLWH around the globe. This could include refining existing interventions to help PLWH and their communities build trust in medical and social service organizations before recommending challenging health-related behavioral changes including medication adherence, dietary and physical activity changes, and decreasing substance use [61,62,64,65]. Our observations clearly suggest individual health is one essential element in the complex web of social and structural factors that constitute social capital and the overall health and wellbeing of PLWH around the globe.

### Limitations

While this study has several advantages, including filling a significant gap in the literature, there are limitations that must be considered by the reader. The first limitation is that we used a convenience sampling method, not random sampling. Therefore, our data are only representative of the samples surveyed and the information cannot be extrapolated to the entire population in any country where the samples were obtained. With the exception of the United States, every country only had one site where data were collected. Therefore, it would be inappropriate to base conclusions about an entire country on a single site, especially when considering the geographic size and demographic diversity of the countries studied (Canada, China, Namibia, and Thailand). However, even though country-level extrapolation is not appropriate, our study is among the first to describe individual-level social capital in some of these sites, which allows for tempered cross-national comparison. An additional limitation may be the modification of the Social Capital Scale by the removal of the 5 work-related items. While this strategy was similar to the one adopted by Onyx and Bullen, it is possible that this strategy could have led to an upward bias in our overall summary statistics. Finally, most of our data collection sites were in the United States and in our summary statistics, the U.S. estimates exerted more weight, leading to an overall U.S. bias in these statistics. These analyses also assume that there is a level of homogeneity among the participants simply because they are all PLWH, which may be an unjustified assumption. We tried to address these concerns by providing the data at both individual site level and at the country level. Additionally, to better determine the influence that country may have had on our results, we explored this issue with multiple regression analyses (Additional file 4: Table S4). These results indicate that, despite the overrepresentation of participants living in the United States, country of origin does not influence our model. Thus, the risk of U.S. bias on results appears to be minimal.

## Conclusions

Social capital is an intriguing and promising concept in public health and a general description of individual-level social capital in specific subpopulations can provide a springboard for future work. As an exploratory study it was not possible to obtain a truly representative sample of international PLWH. We do not provide an exhaustive or general description of PLWH from the study countries, nor to all international PLWH; however, this study gives a limited description of individual-level social capital among international populations living with HIV/AIDS. This study sample came from clinic attendees in the 16 different study locations and may have resulted in a group which is likely more competent and possibly more networked compared to other PLWH. Despite the population not being a representative sample it is informative in creating Social Capital interventions, since those who regularly attend clinics and use AIDS service organizations are also the most likely to take part in the interventions which may be developed from this preliminary study. Research and public health interventions that emphasize increasing total social capital, or aspects of social capital in PLWH could be built upon our descriptive findings. Furthermore, researchers and public health interventionists can be confident that the Social Capital Scale is an appropriate instrument to measure change in perceived individual-level social capital and can be measured in tandem with relevant health outcomes. Finally, the relationship between total social capital and perceived physical and psychological health outcomes in PLWH adds to the growing evidence of relationships between social capital and health. Through continued study of these relationships, we have much to learn about how the perceived social environment influences individual-level health. With enhanced knowledge of the complex relationships in the social environment we may be able to make high-impact changes to improve the health of all.

## Abbreviations

HIV/AIDS: Human immunodeficiency virus/acquired immune deficiency syndrome; PLWH: Person living with HIV; SF: Short form-12; CES-D: Center for epidemiologic studies depression scale; SD: Standard deviation; GED: General equivalency development test; AA: Associate's degree; BS/BA: Bachelors degree in science/arts.

## Competing interests

The authors declare that they have no competing interests.

## Authors' contributions

This work was completed as part of an international research collaborative, and was only possible because of the hard work and commitment by each site investigator. Each author substantially contributed to designing the research protocol, collected data, assisted in the analysis, and in the writing of this manuscript. Therefore, each author made significant contributions to this work outlined below and we want to acknowledge these contributions. Each author has approved of the final version of this manuscript.

ARW conceived the research question and design, collected data, completed the analysis, and interpreted of data, and wrote the first draft of the manuscript. JCP, contributed to developing the research question, study design, collected data and assisted with the analysis and assisted Dr Webel in writing the manuscript. CDR, contributed to developing the research study design, collected data and assisted with the analysis and assisted Dr Webel in writing the manuscript and read and agrees with the final version submitted to *BMC Public Health*. WLH contributed to developing the research study design, collected data and assisted with the analysis and interpretation of the data, and he assisted Dr Webel in writing the manuscript. WTC contributed to developing the research study design, translated the instruments and protocol, collected data and assisted with the analysis and interpretation of the data and she assisted Dr Webel in writing the manuscript. LTV contributed to developing the research study design, collected data and assisted with the interpretation of the data and assisted Dr Webel in writing the manuscript. MRM contributed to developing the research study design, collected data and assisted with the interpretation of the data and assisted Dr Webel in writing the manuscript.PN contributed to developing the research study design, collected data and assisted with the interpretation of the data and assisted Dr Webel in writing the manuscript. KN contributed to developing the research study design, translated the instruments and protocol, collected data and assisted with the interpretation of the data and assisted with the analysis and interpretation of the data and she assisted Dr Webel in writing the manuscript. JK, contributed to developing the research study design, collected data and assisted with the interpretation of the data and assisted Dr Webel in writing the manuscript. ES contributed to developing the research study design, collected data and assisted with the interpretation of the data and assisted Dr Webel in writing the manuscript. JB contributed to developing the research study design, collected data and assisted with the analysis and interpretation of the data and he assisted Dr Webel in writing the manuscript. LE, contributed to developing the research study design, collected data and assisted with the interpretation of the data and assisted Dr Webel in writing the manuscript.SI contributed to developing the research study design, translated the instruments and protocol, collected data and assisted with the analysis and interpretation of the data and she assisted Dr Webel in writing the manuscript. KK contributed to developing the research study design, collected data and assisted with the interpretation of the data and assisted Dr Webel in writing the manuscript. DW contributed to developing the research study design, collected data and assisted with the interpretation of the data and assisted Dr Webel in writing the manuscript. PC contributed to developing the research study design, translated the instruments and protocol, collected data and assisted with the analysis and interpretation of the data and she assisted Dr Webel in writing the manuscript. MJ contributed to developing the research study design, collected data and assisted with the analysis and interpretation of the data and he assisted Dr Webel in writing the manuscript. CP, contributed to developing the research study design, translated the instruments and protocol, collected data and assisted with the analysis and interpretation of the data. She assisted Dr Webel in writing the manuscript. IBC contributed to developing the research study design, collected data and assisted with the interpretation of the data and assisted Dr Webel in writing the manuscript. JV contributed to developing the research study design, collected data and assisted with the interpretation of the data and assisted Dr Webel in writing the manuscript. RAS contributed to collecting the data, interpreting the data, and assisting Dr. Webel in writing the manuscript. All authors read and approved the final manuscript.

## Pre-publication history

The pre-publication history for this paper can be accessed here:

http://www.biomedcentral.com/1471-2458/12/188/prepub

## Supplementary Material

Additional file 1 **Table S1**. Means, Medians, and Cronbach's α for the Social Capital Survey by Site. Click here for file

Additional file 2 **Table S2**. Individual Factor Loadings. Click here for file

Additional file 3 **Table S3**. Bivariate Correlation Coefficients between Social Capital and Mental health Variables in HIV+ Adults. Click here for file

Additional file 4 **Table S4**. Relationship between Individual Social Capital in HIV + Adults and variables consistent with Social Action Theory (n = 1,082)^1^. Click here for file
