# Supplementary material for: A cross-sectional description of social capital in an international sample of persons living with HIV/AIDS (PLWH)
Source: BMC Public Health. 2012 Mar 13;12:188. doi: 10.1186/1471-2458-12-188 (PMC3352053; doi:10.1186/1471-2458-12-188)
Supplement: Additional file 1 — Table S1. Means, Medians, and Cronbach's α for the Social Capital Survey by Site. [file 1471-2458-12-188-S1.DOC]

Supplemental Table 1: Means, Medians, and Cronbach’s α for the Social Capital Survey by Site

| Site | Total sample size | Mean Social Capital (SD) | Median Social Capital | Total Social Capital Scale  Cronbach’s α |
| --- | --- | --- | --- | --- |
| San Francisco, CA | 300 | 2.63 (0.55) | 2.61 | 0.89 |
| Boston, MA | 200 | 2.67 (0.56) | 2.71 | 0.88 |
| Seattle, WA | 200 | 2.58 (0.53) | 2.61 | 0.90 |
| Shanghai, China | 107 | 2.56 (0.49) | 2.55 | 0.90 |
| Vancouver, Canada | 100 | 2.61 (0.52) | 2.58 | 0.88 |
| Bangkok, Thailand | 100 | 2.61 (0.55) | 2.65 | 0.87 |
| San Juan, PR | 100 | 2.81 (0.48) | 2.77 | 0.84 |
| Chicago, IL | 95 | 2.69 (0.57) | 2.65 | 0.90 |
| Cleveland, OH | 150 | 2.61 (0.52) | 2.56 | 0.88 |
| Wilmington, NC | 100 | 2.78 (0.53) | 2.74 | 0.87 |
| Durham, NC | 100 | 2.84 (0.58) | 2.87 | 0.90 |
| New York, NY | 100 | 2.70 (0.61) | 2.63 | 0.90 |
| Newark, NJ | 100 | 2.68 (0.43) | 2.69 | 0.82 |
| Windhoek,Namibia | 102 | 2.95 (0.66) | 2.68 | 0.93 |
| Corpus Christi, TX | 94 | 2.68 (0.58) | 2.71 | 0.90 |
| Harlington, TX | 115 | 2.64 (0.47) | 2.66 | 0.84 |
| **Total for all sites** | **1,963** | **2.68 (0.55)** | **2.65** | **0.88** |
