# Supplementary material for: A cross-sectional description of social capital in an international sample of persons living with HIV/AIDS (PLWH)
Source: BMC Public Health. 2012 Mar 13;12:188. doi: 10.1186/1471-2458-12-188 (PMC3352053; doi:10.1186/1471-2458-12-188)
Supplement: Additional file 2 — Table S2. Individual Factor Loadings. [file 1471-2458-12-188-S2.DOC]

Supplemental Table 2: Individual Factor Loadings

| Item | *Factor and Loading* | *Onyx and Bullen Factor* |
| --- | --- | --- |
| 1. Do you feel valued by society?^1^ | Friends and Family Connections (0.42) | Value of Life |
| 2. If you were to die tomorrow, would you be satisfied with what your life has meant? ^1^ | Friends and Family Connections (0.37) | Value of Life |
| 3. Have you ever picked up other people’s trash in a public place? ^1^ | Non-loading (0.65) | Social Agency or Proactivity in a Social Context |
| 4. Some say that by helping others you help yourself in the long run. Do you agree? ^1^ | Non-loading (0.36) | Value of Life |
| 5. Do you help out a local group as a volunteer? | Participation in the Local Community (0.59) | Participation in the Local Community |
| 6. Do you feel safe walking down your street after dark? | Feelings of Trust and Safety(0.53) | Feelings of Trust and Safety |
| 7. Do you agree that most people can be trusted? | Feelings of Trust and Safety (0.47) | Feelings of Trust and Safety |
| 8. If someone’s car breaks down outside your house, do you invite them into your home to use the phone? | Neighborhood Connections (0.60) | Feelings of Trust and Safety |
| 9. Can you get help from friends when you need it? ^1^ | Friends and Family Connections (0.40) | Neighborhood Connections |
| 10. Does your area have a reputation for being a safe place? | Feelings of Trust and Safety (0.68) | Feelings of Trust and Safety |
| 11. If you were caring for a child and needed to go out for a while, would you ask a neighbor or help? | Neighborhood Connections (0.68) | Neighborhood Connections |
| 12. Have you visited a neighbor in the past week? | Neighborhood Connections (0.62) | Neighborhood Connections |
| 13. Have you attended a local community event in the past 6 months (e.g., church, school concert, craft sale)? ^1^ | Non-loading (0.46) | Participation in the Local Community |
| 14. Are you an active member of a local organization or club (e.g., sport, craft, social club)? | Participation in the Local Community (0.64) | Participation in the Local Community |
| 15. Does your local community feel like home? | Feelings of Trust and Safety (0.32) | Feelings of Trust and Safety |
| 16. In the past week, how many phone conversations have you had with friends? | Friends and Family Connections (0.67) | Family and Friends Connections |
| 17. How many people did you talk to yesterday? | Friends and Family Connections (0.65) | Family and Friends Connections |
| 18. Over the weekend do you have lunch/dinner with other people outside your household? | Friends and Family Connections (0.52) | Family and Friends Connections |
| 19. Do you go outside your local community to visit your family? ^1^ | Friends and Family Connections (0.62) | Social Agency or Proactivity in a Social Context |
| 20. When you go shopping in your local area are you likely to run into friends and acquaintances? ^1^ | Friends and Family Connections (0.53) | Neighborhood Connections |
| 21. If you need information to make a life decision, do you know where to find that information? ^1^ | Friends and Family Connections (0.43) | Social Agency or Proactivity in a Social Context |
| 22. In the past 6 months, have you done a favor for a sick neighbor? | Neighborhood Connections (0.39) | Neighborhood Connections |
| 23. Are you on a management committee or organizing committee for any local group or organization? | Participation in the Local Community (0.78) | Participation in the Local Community |
| 24. In the past 3 years, have you ever joined a local community action to deal with an emergency? | Participation in the Local Community (0.79) | Participation in the Local Community |
| 25. In the past 3 years have you ever taken part in a local community project? | Participation in the Local Community (0.80) | Participation in the Local Community |
| 26. Have you ever been part of a project to organize a new service in your area (eg, youth club, child care, recreation for disabled)? | Participation in the Local Community (0.77) | Participation in the Local Community |
| 27. If you disagree with what everyone else agreed on, would you feel free to speak out? ^1^ | Tolerance and Diversity (0.56) | Social Agency or Proactivity in a Social Context |
| 28. If you have a dispute with your neighbors (eg, over music or animals) are you willing to seek mediation? ^1^ | Tolerance and Diversity (0.57) | Social Agency or Proactivity in a Social Context |
| 29. Do you think that diversity makes life in your area better? | Tolerance and Diversity(0.78) | Tolerance of Diversity |
| 30. Do you enjoy living among people of different life styles? | Tolerance and Diversity (0.76) | Tolerance of Diversity |
| 31. If a stranger, someone different, moves into your street, would they be accepted by the neighbors? | Tolerance and Diversity (0.63) | Tolerance of Diversity |

^1^ if different from Bullen and Ony,x=11
