# Supplementary material for: A cross-sectional description of social capital in an international sample of persons living with HIV/AIDS (PLWH)
Source: BMC Public Health. 2012 Mar 13;12:188. doi: 10.1186/1471-2458-12-188 (PMC3352053; doi:10.1186/1471-2458-12-188)
Supplement: Additional file 3 — Table S3. Bivariate Correlation Coefficients between Social Capital and Mental health Variables in HIV+ Adults. [file 1471-2458-12-188-S3.DOC]

Supplemental Table 3: Bivariate Correlation Coefficients between Social Capital and Mental health Variables in HIV+ Adults

| Country | Sample Size | Mental Health Quality of Life^1^  (p-value) | Depression CES-D^1^  (p-value) | Anxiety ^2^  (p-value) | IVDU^2^  (p-value) | Alcohol Use^2^  (p-value) |
| --- | --- | --- | --- | --- | --- | --- |
| Canada | 100 | −0.03 (0.81) | −0.01 (0.90) | −0.12 (0.33) | 0.11 (0.37) | −0.11 (0.36) |
| China | 107 | 0.08 (0.43) | −0.005 (0.96) | −0.15 (0.14) | −0.06 (0.57) | 0.00 (0.95) |
| Namibia | 102 | 0.005 (0.96) | 0.08 (0.44) | −0.12 (0.31) | 0.17 (0.13) | 0.23 (0.05) |
| Puerto Rico | 100 | 0.14 (0.25) | 0.10 (0.37) | −0.13 (0.25) | −0.11 (0.35) | −0.22 (0.06) |
| Thailand | 100 | 0.15 (0.24) | 0.14 (0.23) | −0.18 | 0.11 | 0.18 |
| United States | 1,454 | 0.01 (0.24) | −0.02 (0.61) | −0.01 (0.64) | 0.02 (0.42) | 0.02 (0.44) |
| Total | 1,963 | 0.02 (0.37) | −0.002 (0.95) | −0.05 (0.08) | 0.03 (0.27) | 0.02 (0.44) |

^1^Distribution of the data indicated that a Pearson product–moment correlation coefficient was the appropriate bivariate correlation statistic. We ran this analysis.

^2^Distribution of the data indicated that a Spearman's rank correlation coefficient was the appropriate bivariate correlation statistic. We ran this analysis
