# Supplementary material for: A cross-sectional description of social capital in an international sample of persons living with HIV/AIDS (PLWH)
Source: BMC Public Health. 2012 Mar 13;12:188. doi: 10.1186/1471-2458-12-188 (PMC3352053; doi:10.1186/1471-2458-12-188)
Supplement: Additional file 4 — Table S4. Relationship between Individual Social Capital in HIV + Adults and variables consistent with Social Action Theory (n = 1,082)1. [file 1471-2458-12-188-S4.DOC]

**Supplemental Table 4: Relationship between Individual Social Capital in HIV+ Adults and variables consistent with Social Action Theory (n=1,082)^1^**

| **Model 1** | ***β*** | **95% CI** | ***p- value*** | ***F***  **(df)** | ***AdjustedR^2^*** |
| --- | --- | --- | --- | --- | --- |
| Age | 0.006 | 0.003-0.009 | <0.001 |  |  |
| Race | −0.048 | −0.068- -0.028 | <0.001 |  |  |
| Education level | 0.071 | 0.044-0.098 | <0.001 |  |  |
| Recent CD4 count | 0.09 | 0.019-0.155 | 0.012 |  |  |
| Self-reported psychological condition | 0.035 | 0.020-0.050 | <0.001 |  |  |
| Self-reported social support | 0.065 | 0.052-0.079 | <0.001 |  |  |
| Criminalization of HIV | −0.071 | −0.130- -0.013 | 0.017 |  |  |
| Constant | 1.669 | 1.147-1.868 | <0.001 | 50.45  (7, 1074) | 0.24 |
| Model 2 ^2^ |  |  |  |  |  |
| Age | 0.006 | 0.003-0.009 | 0.001 |  |  |
| Race | −0.048 | −0.068- -0.028 | <0.001 |  |  |
| Education level | 0.071 | 0.043-0.098 | <0.001 |  |  |
| Recent CD4 count | 0.09 | 0.019-0.155 | 0.012 |  |  |
| Self-reported psychological condition | 0.035 | 0.020-0.050 | <0.001 |  |  |
| Self-reported social support | 0.065 | 0.052-0.079 | <0.001 |  |  |
| Criminalization of HIV^3^ | −0.081 | −0.150- -0.012 | 0.02 |  |  |
| Country ^4^ | −0.006 | - 0.026- 0.015 | 0.602 |  |  |
| Constant | 1.691 | 1.147-1.907 | <0.001 | 44.15  (8, 1073) | 0.24 |

CI = confidence interval, df = degrees of freedom ^1^Variable selection based on variables in the Social Action Theory; ^2^Controlling for country; ^3^ State or Country level policy exists that criminalizes HIV transmission; ^4^ Country was dummy coded to explore the influence of varying sample sizes among the different country sites.
